# Correlational analysis of occupational accidents and the safety policies in the Chinese coal mining industry from 2008 to 2021

**DOI:** 10.1038/s41598-024-54241-3

**Published:** 2024-02-14

**Authors:** Anyu Zhu, Qifei Wang, Haolin Liu, Hongqing Zhu, Yun Lei

**Affiliations:** 1 School of Emergency Management and Safety Engineering, China University of Mining and Technology (Beijing), Beijing, 100083 China; 2 School of Mechanical-Electronic and Vehicle Engineering, Beijing University of Civil Engineering and Architecture, Beijing, 102616 China; 3 Shenyang Research Institute, China Coal Technology Engineering Group, Liaoning, 110000 China

**Keywords:** Accident statistics, Coal mine accidents, Correlation characteristic, Safety policy, Environmental sciences, Environmental social sciences

## Abstract

This study investigates the correlation between previous coal mine safety policies and accidents in China. Data on coal mine accidents and government regulatory information from 2008 to 2021 are collected. The characteristics of coal mine accidents are analyzed, and safety policy indexes are identified. An ordinary least squares (OLS) regression model is established to quantitatively analyze the correlation between accidents and safety policy. The study finds that safety policies have some impact on accident occurrence in coal mines. Although there has been a decrease in accidents and deaths over time, higher mortality rates are observed during periods of increased production intensity and on weekends. Gas accidents are the most common, followed by roof and flood accidents. The study concludes that national safety policies with wider coverage and a stronger system are effective in preventing accidents, but caution should be exercised to avoid reduced vigilance with decreasing death rates.

## Introduction

Nowadays, coal, as one of the main energies in the world, has played an indispensable role in the survival and development of human society. Especially in modern industry, coal plays an important role in energy, chemical, metallurgy, machinery, light textile, food, and other industries. However, because of the harsh working conditions, the death toll of coal mine accidents is always high due to various reasons, such as mechanical failure^1^, gas explosion^2^, weather reasons^3^, poor safety management of enterprises^4^ and roof accidents. To reduce the death toll of coal mine accidents, various countries have promulgated a series of policies and management methods combined with the actual situation^5^ and achieved certain effects. Whether and how coal mine production policies can directly affect the number of coal mine production accidents and deaths have always been the focus of research by experts and scholars in the field of coal mine safety, which is also of great significance to the formulation of relevant coal mine policies in the future.

In the field of safety science, many scholars have conducted research on the effect of the implementation of policies on various accidents. David et al.^6^focused on the results of policies to encourage cycling in some European cities and found that they could significantly reduce the number of people killed in road accidents each year and reduce carbon dioxide emissions. Jan et al.^7^ conducted research on the relationship between the safety management practice systems and objective safety statistics. The results show that there was a significant negative correlation between the existence of personal safety management measures, their composite measures, the accident rate, the emotional and cognitive input level of safety-oriented employees was significantly negatively correlated with the accident rate. In addition, another study found that the appropriate security policies can also protect the rights and interests of workers^8^. Washington found that due to the related policy is not perfect, some injured workers did not record for industrial injury. This will not only affect the injured workers not getting the medical care they need or deserve compensation, but also result in the persistence of unhealthy and unsafe environments^9^. After the implementation of the OHSAS 18001 standard, by investigating the impact of the policy on enterprises, it can be found that safety standards are a critical strategic component for enterprises, not only to improve safety conditions in the workplace but also to help create a competitive advantage and consolidate business operations^10^. It can be seen that security policy is an indispensable part of all walks of life, and its existence can effectively guarantee the safety of residents' lives and property.

Some researchers have also researched on the influence of safety policies on coal mine accidents. They have compared the results of coal mine safety policies implemented in the past with the number of coal mine accidents before the implementation. The results show that there is a linear relationship between the number of deaths in coal mine accidents and the number of safety policies^11,12^, and external policies have a greater impact on enterprise safety^13–15^. Implementation of relevant policies^16–18^ and measures for safety management^19–21^ plays a positive role in the prevention of coal mine production accidents. Adopting correct and appropriate policies can effectively reduce the risk of coal mine production accidents, and a sound safety production management system is necessary for the production of enterprises^22^. Wang et al.^11^ quantitatively analyzed the impact of safety policy types on the prevention of work-related accidents. The result shows that when the safety environment is affected by positive safety policies, the mortality rate per 100 million people shows a significant negative change, that is, the implementation of safety policies has a significant inhibitory effect on the number of coal mine accident deaths. In addition, they find out that different levels of policies have different impacts on the production safety environment. The impact of policies at the national level is much greater than that of local standard policies, so national security policies are more important in the formulation of the production safety environment. For developing countries with imperfect security policies, it is necessary to increase the number of security policies and improve the efficiency of their implementation. For countries with well-developed or even strict safety systems, the regulation can be appropriately relaxed. For enterprises, it can be combined flexibly without accidents, to improve the production vitality of enterprises. A similar study on the influence of safety policy on accident occurrence is also conducted by Ke and Wang^12^, and the results are consistent with previous experimental conclusions. From 2003 to 2018, due to the introduction of a series of work safety policies in China, the death rate per million tons of coal in China decreased from 3.724 in 2003 to 0.093 in 2018, showing an alarmingly rapid downward trend, which played an important role in reducing coal mine accidents in China. The results show that the safe implementation of safety policies is an important measure to ensure the safety of production activities and has important guiding significance for high-risk industries, accident-prone areas, and areas with imperfect policies. Recently, there have been many coal mine accidents in China, according to the report from the official website of National Mine Safety Administration of China, there were a total of 168 coal mine accidents and caused 245 deaths in China in 2022. We should draw lessons from past accidents^23^. The research shows that compared with foreign developed countries, the development of coal mining enterprises in China has not reached the equilibrium state, and there is still room for further development^24–26^. In some aspects, China still lacks relevant policies^27^. Enterprise managers and workers have insufficient awareness of safety education^28^ and insufficient safety investment^29–31^. Therefore, relevant policies should be formulated to improve the overall safety of production work. Enterprises also need to implement relevant safety policies, strengthen safety training and education for coal mine employees, and learn from the experience and lessons of mining enterprises in developed countries.

Accordingly, the occurrence of coal mine accidents is the result of the joint action of many factors, among which various safety supervision and management policies are significantly related to the occurrence of coal mine accidents. Strict coal mine safety policy can play a good role in protecting coal mine production safety. In addition, the implementation of correct and appropriate policies can make the social production environment play a virtuous circle, which can not only effectively ensure the safety of workers but also create better economic benefits for the society. The existing research is still based on qualitative analysis, quantitative analysis is not deep enough. At present, there is less research on coal mine accidents related to safety production policies, and it is evident that with the promulgation of various policies in recent years, the number of safety production accidents and deaths has significantly decreased. However, there are also many types and quantities of policies, and the actual role of each type of policy in reducing the number of coal mine accidents is difficult to define. And, there is also less research that can quantitatively analyze this problem. Therefore, the authors think that quantitative analysis is insufficient. Therefore, this paper studied the characteristics of coal mine accidents in China based on the collecting data on coal mine accidents and regulatory information disclosed by the government in China from 2008 to 2021. In this paper, the influence of the safety policies on accidents is analyzed qualitatively and quantitatively, and a mathematical model is established to accurately describe the accident data. The finding of this study can provide a theoretical reference for the study of the factors affecting work-related accidents in coal mine industry and other industries, and the formulation of safety policies in the future.

## Materials and methods

### Coal mine accident characteristics in China

The data used in this study comes from the Ministry of Emergency Management of the People’s Republic of China (State Administration of Work Safety), and the Nation Mine Safety Administration.

**Figure 1:**
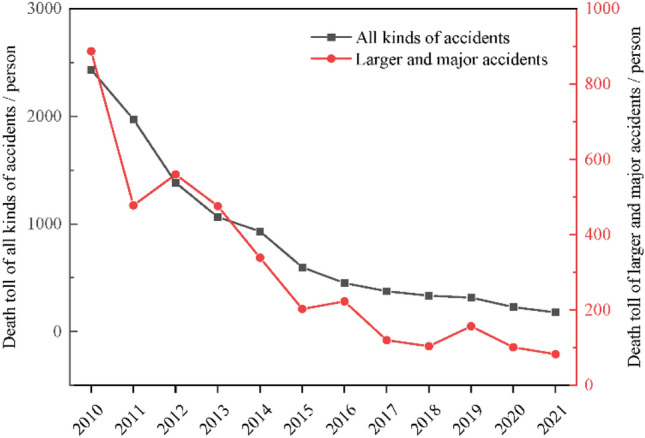
The overall trend of major accidents and all accidents.

According to the "Regulations on Reporting and Investigating Production Safety Accidents" issued by the Chinese government, accidents with three or more deaths are classified as major or more serious accidents, while accidents with less than three deaths are classified as general accidents. Major or more serious accidents are investigated by higher-level government agencies, which are usually national or provincial coal mine safety supervision and management agencies. And we can get detailed accidents information with 3 or more deaths from the website of National Mine Safety Administration of China. For other accidents, government departments will disclose the total number of deaths per year. Thus, the coal mine accidents with 3 or more death were selected for the following analysis in this paper and the total number of these accidents is 2563. The number of accidents with 3 or more fatalities and all death accidents was plotted as time series, as shown in Fig. 1. Figure 1 shows that the overall trend of major accidents and all accidents with time is similar. Therefore, the number of accidents with 3 or more fatalities can reflect the law of coal mine accidents, which can be helpful to find the effect of safety policy on the prevention of coal mine accidents.

Coal mine safety policy information used in this study comes from the National Database of Laws and Regulation, the Ministry of Emergency Management of the People’s Republic of China (State Administration of Work Safety), the Nation Mine Safety Administration, the service platform of industry standard, and the website of the Chinese Government. Then, policies from 2008 to 2021 are selected for analysis.

**Figure 2:**
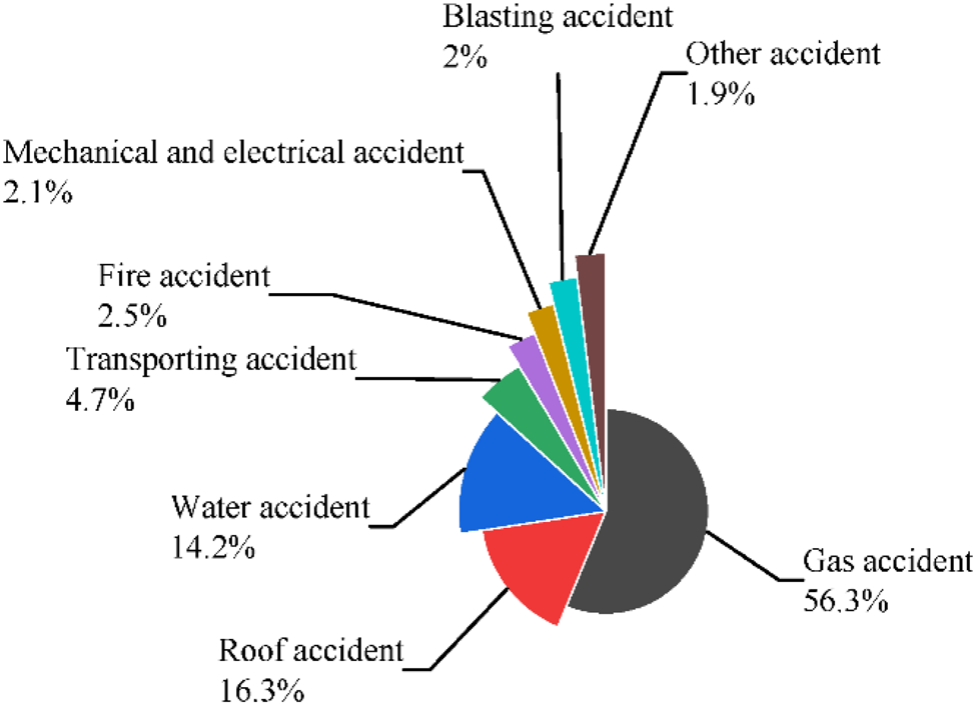
Distribution of accident type in all the coal mine accidents from 2010 to 2020.

Figure 2 shows the number of different accident types. From Fig. 2, gas accidents account for more than half of major and above accidents. Gas may induce explosion, poisoning, fire, and other accidents. Gas accidents are the most dangerous accident type in coal mines. Roof and flood accidents have also been types with a high frequency, which are only lower than gas accidents.

### Coal mine accident characteristics in America

**Figure 3:**
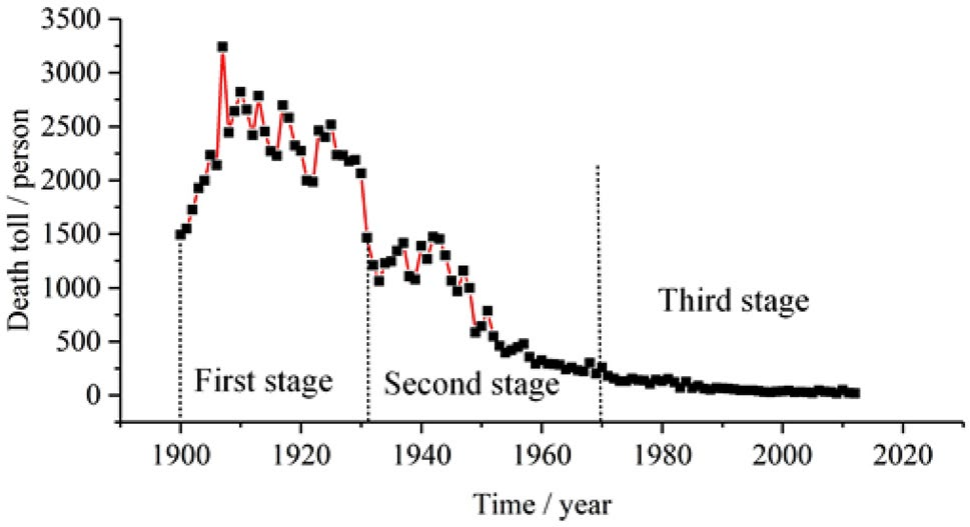
The death toll of coal accidents in America from 1900 to 2012.

After research, we got the relationships between the death toll of coal accidents and the safety policies in America. The death toll of coal accidents in America from 1900 to 2012 is shown as Fig. 3.

It can be seen that the death toll in America shows obvious stage characteristics. The first stage is the high incidence period of coal accidents which is from 1900 to 1930. The second stage is the rapid decline period of coal accidents which is from 1931 to 1970. The third stage is the stable status period which is from 1971 to 2012. In first stage, to strengthen the safety management of coal mines, the United States federal government established the Bureau of Mines through organizational law in 1910. The Mining Bureau, together with various states, has successively formulated some regulations and accident investigation and handling laws for coal mine safety production, as well as the development of coal mine ventilation equipment and safety instruments such as gas detectors. The implementation of these safety measures has significantly promoted the improvement of coal mine safety conditions.

In the second stage, the United States issued coal mine safety regulations in 1941, requiring the establishment of a coal mine safety supervision agency and the deployment of coal mine safety inspectors. Federal regulations on coal mine safety were also enacted. These policies reduced the occurrence of coal mine accidents greatly. The death toll of coal mine accidents reduced from 1266 in 1941 to 260 in 1970. In the third stage, these policies continuously achieving good results and kept the death toll of coal mine accidents in a stable and low level. Above all, no matter in China or America, the safety policies can affect the coal mine accidents obviously. Some safety policies and relevant measures reduced the number of coal mine accidents effectively.

### Comparison between coal mine and construction industry accidents

**Figure 4:**
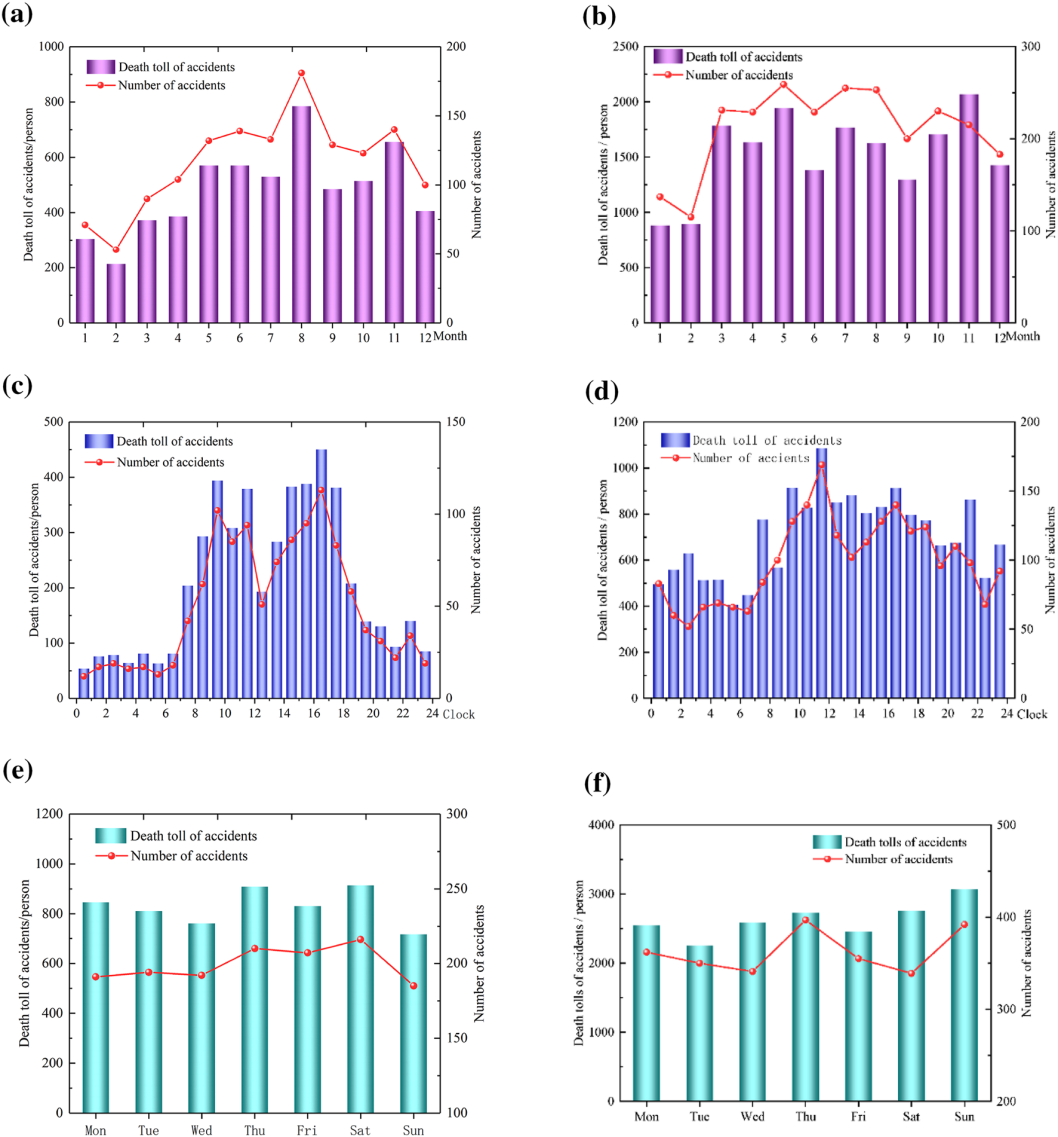
Comparison between coal mine and construction industry accidents.

Figure 4 shows the comparison between coal mine and construction industry accidents. It can be seen, for the coal mine, the death number has no obvious difference from the day in a week, the month in a year and the clock in a day. However, we also obtained the death number data of the construction industry. We found that the death number in construction industry shows obvious difference from the day in a week, the month in a year and the clock in a day. For the construction industry, the August has the highest death toll in a year, it’s because the August has the highest outdoor temperature in a year in China and the worker mostly works outdoor in construction industry, the workers are easily affected by the high temperature in August which causes the high number of accidents and death. But for coal mine, workers work in underground tunnel which is indoor environment, the workers are not affected by the outdoor high temperature, the work environment basically don’t change in a year, that’s the reason why the accident number have no big difference in different months in a year. For the construction industry, the 17:00 has the highest death toll in a day, it’s because the workers only work in daylight in construction industry, at 17:00, the workers are very tired after longtime working which also easily causes the high number of accidents and death. But for coal mine, the workers obey the three-shift work system, the times that workers become tired are not unified in different coal mines. For the days in a week, the death tolls don’t have big difference in both construction industry and coal mines. It’s because workers in both construction industry and coal mines need to work as normal in weekend.

### Identification of index of safety policy

**Figure 5:**
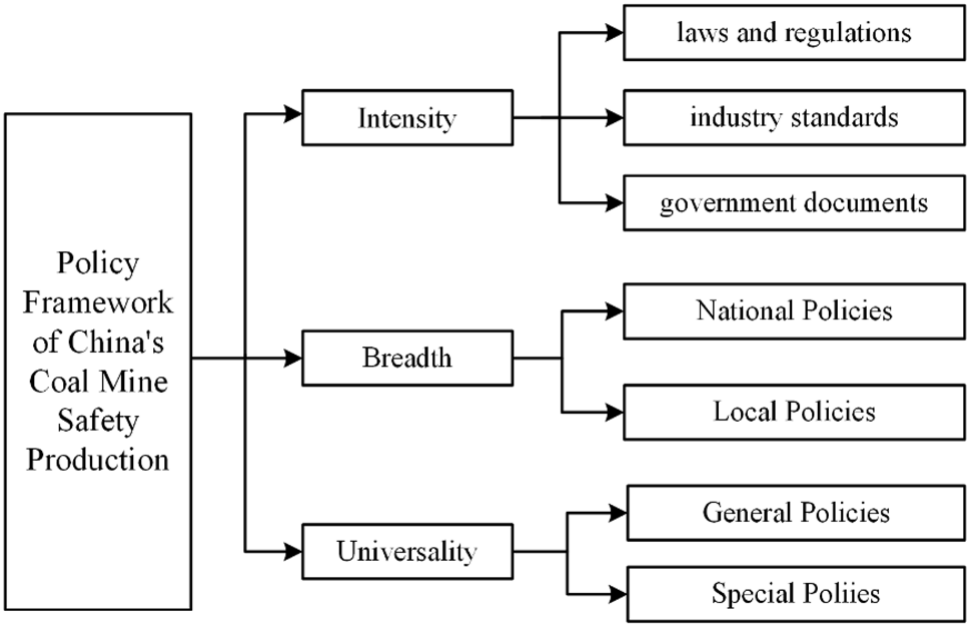
The indexes of coal mine safety policies in China.

In this section, a classified analysis of safety policies is conducted and indexes of coal mine safety policy are then identified, which are used for the quantitative analysis of the correlation characteristics between coal mine accidents and safety policies. Three indexes are identified in this study, including intensity, breadth, and universality. Figure 5 shows the indexes of coal mine safety policies in China.

According to the intensities(I), China's coal mine safety policies are classified into laws and regulations, industry standards and government documents. Laws and regulations refer to the safety laws and regulations in China. Industry standards refers to safety specifications and standards issued by industry departments. Government documents, that is documents related to safety specifications issued by the safety supervision department and the State Council. Both laws and regulations, as well as government documents, are formulated by the Chinese government, but the binding force of laws and regulations is stronger than that of government documents. Safety laws and regulations in the coal mining sector are strictly enforced by all coal mines in China during production, and the number of such documents is relatively small. Government documents, on the other hand, typically serve as supplements to laws and regulations, providing specific requirements or guidance on safety issues in production. Their mandatory nature is weaker compared to laws and regulations.

**Figure 6:**
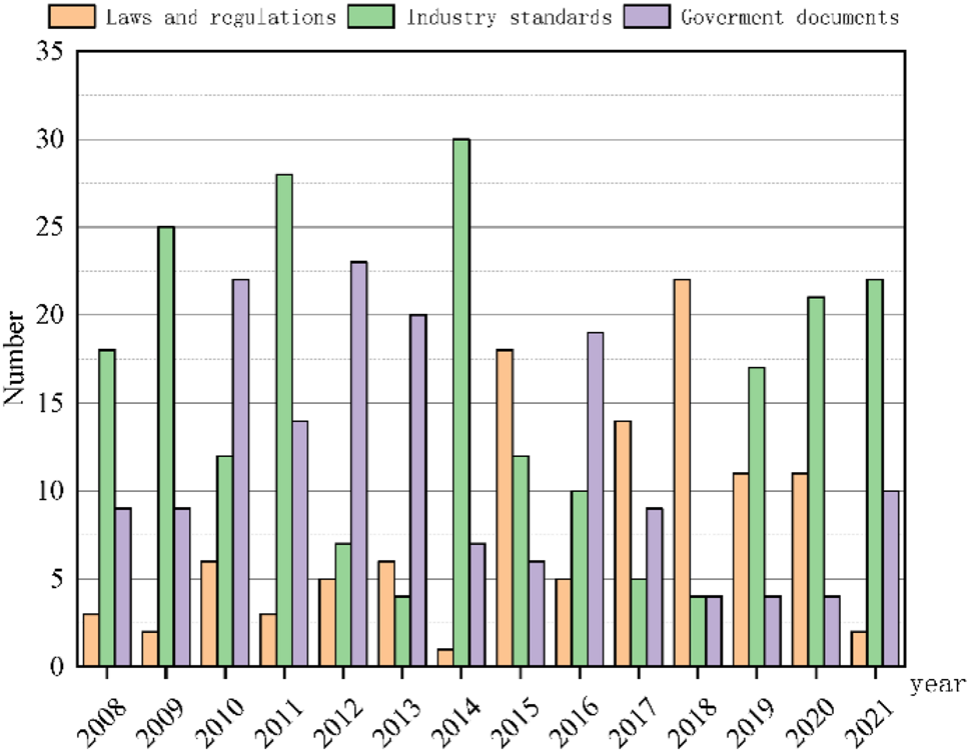
The number of safety policies with different intensities from 2008 to 2021.

**Figure 7:**
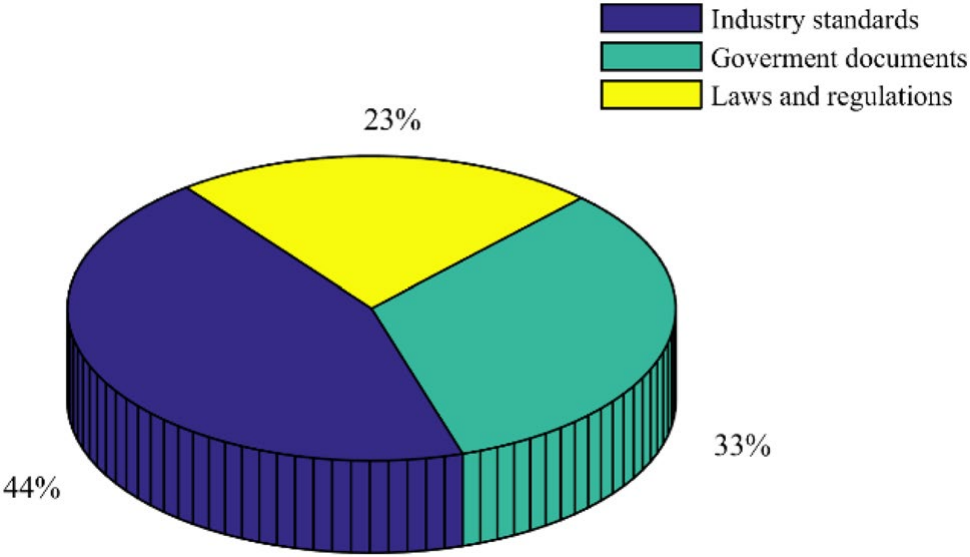
The distribution of safety policies with different intensities.

Figure 6 shows the number of safety policies with different intensities from 2008 to 2021. From Fig. 5, in 2018, the most laws and regulations were passed. Similarly, the largest number of industrial standards and government documents were passed in 2014 and 2012 respectively. Figure 7 shows the distribution of safety policies with different intensities. It can be seen from Fig. 6 that the number of industrial standards is the largest, followed by government documents, and the number of laws and regulations is the smallest.

According to the breadth(B), China's coal mine safety policies are classified into national policies and local policies. According to the universality(U), the policies are classified into general policy and special policy.

With the aspect of legal strength, the legal effect of laws and regulations, industry standards and government documents are gradually reduced, and are respectively given 3, 2 and 1, as shown in Table 1. From the perspective of the coverage of policies, there are two levels: national and local, given the score 2 and 1 respectively. In terms of the scope of application, the general policy has a wider scope of application with greater flexibility, which is applicable to many industries, such as mining, chemical. While the special policy has a relatively small scope of application, which is only applicable to the coal mining industry. They are thus given the score 2 and 1 respectively.

**Table 1:** Coal mine policy indicators.

Indicators	Intensity	Breadth	Universality
Level	Laws and regulations 3 Industry standards 2 Government documents 1	National policies 2 Local policies 1	General policies2 Special poliies1

**Figure 8:**
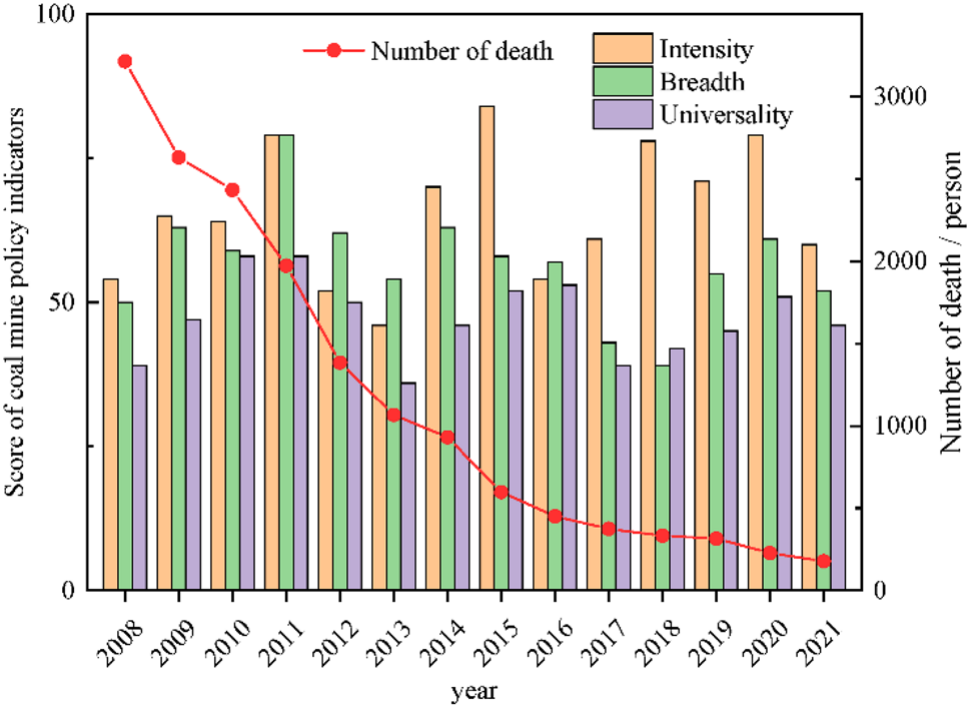
The trend of coal mine safety policy indicators and the number of deaths from 2008 to 2021.

Figure 8 shows the trend of coal mine safety policy indicators from 2008 to 2021. From Fig. 7, it can be seen that the number of fatalities (Y) in accidents has a significant downward trend over time. In addition, in 2011, the intensity and breadth reached a peak, indicating that the large number, high importance and wide coverage of the coal mining policies formulated that year.

## Results and discussion

### OLS regression analysis

In this section, an ordinary least squares (OLS) regression model is established to conduct a quantitative analysis for the correlation characteristics between coal mine accidents and safety policy. The model can not only explain the law of accidents, but also predict the occurrence of coal mine accidents in China. After establishing model according to the quantitative characteristics of safety policies, the model was tested and revised by model fitting and significance test, heteroscedasticity test, and autocorrelation test. Finally, the quantitative relationship between the number of safety production policies and coal mine accidents is analyzed according to the revised regression model.

#### Establishment of OLS regression model

**Figure 9:**
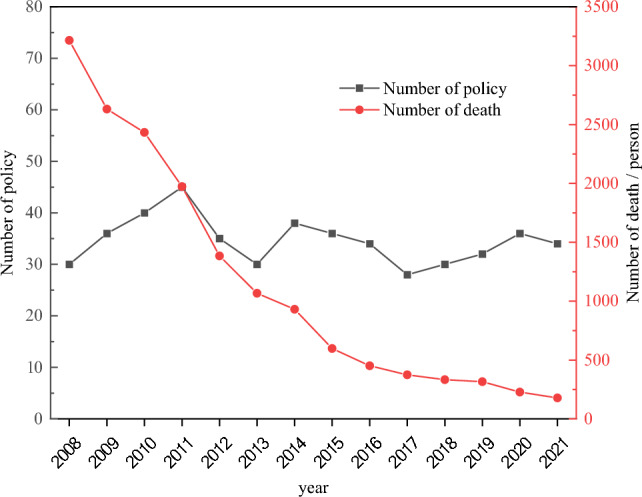
The number of coal mine safety policies and accidents from 2008 to 2021.

Figure 9 shows the number of coal mine safety policies and accidents from 2008 to 2021. It can be seen that the number of policies has been hovering around 35, with repeated fluctuations, but the death toll has steadily declined. It is thus speculated that the implementation of coal mine safety policies will have some impact on the occurrence of accidents. Although the number of policies from 2011 to 2021 did not significantly increase, the mortality rate decreased. This can be attributed to two main factors. Firstly, as depicted in Fig. 8, laws and regulations with greater mandatory intensity and influence, enacted after 2011, constituted a higher proportion. Secondly, the effects of policy implementation typically exhibit a time lag, which will be further discussed later in this article. While the most policies were enacted in 2011, their noticeable impact on reducing accident-related fatalities occurred in the subsequent years.

From Fig. 9, three obvious turning points occurred in 2011, 2013, and 2017 in the trend for the number of safety policies. If we directly segment the data used in the regression analysis, then the sample size and accuracy will be reduced. The virtual variables D1, D2, and D3 are thus created to indicate the three turning points in 2011, 2013, and 2017.

1 $$D_{1} = \left\{ \begin{gathered} 0, \quad 2008 \le t \le 2011 \hfill \\ 1, \quad 2011 < t \le 2021 \hfill \\ \end{gathered} \right.,$$

2 $$D_{2} = \left\{ \begin{gathered} 0, \quad 2008 \le t \le 2013 \hfill \\ 1,\quad 2013 < t \le 2021 \hfill \\ \end{gathered} \right.,$$

3 $$D_{3} = \left\{ \begin{gathered} 0, \quad 2008 \le t \le 2017 \hfill \\ 1, \quad 2017 < t \le 2021 \hfill \\ \end{gathered} \right.,$$

Let Y_t_ be the death toll of coal mine accidents in year t and X_t_ be the number of safety policies in year t. The initial model is thus given by Eq. (4).

4 $$\begin{aligned} Y_{t} & = C + a_{1} X_{t} + a_{2} D_{1} + a_{3} D_{2} + a_{4} D_{3} \hfill \\ & \quad + a_{5} (X_{t} - X_{2011} )D_{1} + a_{6} (X_{t} - X_{2013} )D_{2} \hfill \\ & \quad + a_{7} (X_{t} - X_{2017} )D_{3} + u, \hfill \\ \end{aligned}$$

where C is the intercept, $a_{1}$ to $a_{7}$ are the regression coefficients, X_2011_ is the number of safety policies in 2011, namely 45, X_2013_ is the number of safety policies in 2013, 30 in total, X_2017_ is the number of safety policies in 2017, that is, 28, and u is the random error term.

#### Model test and revise

Table 2 shows the results of model fitting and significance test for the OLS regression model using Eviews10. As shown in Table 2, regression coefficient R2 and revised regression coefficient R2* are greater than 0.9, indicating that the fitting effect of model is well and the test is eligible for test. The established model is thus tenable.

**Table 2:** The results of model fitting and significance test for initial model and revised model.

Model	Goodness of fit		Significance test		C		X		D_1_	
	R_2_	R_2_*	F	P	t	P	t	P	t	P
Initial	0.995	0.989	165.571	0.000	15.040	0.000	− 8.253	0.000	0.100	0.923
Revised	0.994	0.991	354.951	0.000	18.060	0.000	− 10.31	0.000	–	–

Model	D_2_		D_3_		(X_t_-X_2011_)D_1_		(X_t_-X_2013_)D_2_		(X_t_-X_2017_)D_3_	
	t	P	t	P	t	P	t	P	t	P
Initial	− 5.025	0.002	0.44728	0.670	4.514	0.004	− 0.468	0.656	− 2.513	0.046
Revised	− 9.717	0.000	–	–	18.728	0.000	–	–	− 5.222	0.000

The significance t-test of each coefficient shows that the P values of D1, D3 and (Xt-X2013) D2 were greater than 0.1. They were thus eliminated and the revised model is given by Eq. (5). Table 2 also shows the results for revised model. From Table 2, the revised regression coefficient higher than that before modify and F and t tests are more significant. Therefore, the revised model (Eq. (5)) is superior to the original one.

5 $$\begin{aligned} Y_{t}^{ * } & = 5721.6752 - 84.0447X_{t} - 730.3147D_{2} \hfill \\ & \quad + 138.9355(X_{t} - 45)D_{1} - 60.7415(X_{t} - 30)D_{3} \hfill \\ \end{aligned}$$

Table 3 shows the results of heteroscedasticity test and autocorrelation test for the revised model. White heteroscedasticity test is conducted for the revised model, assuming that there is no heteroscedasticity for the random error term u. It can be seen from Table 3 that the concomitant probability P of heteroscedasticity test is 0.0972, which is greater than 0.05. The hypothesis is thus accepted, which is the heteroscedasticity of the model does not exist at the 5% level of significance. Similarly, LM (Lagrange constant) test method is used for the revised model, and the lag order is selected as the first order, assuming that the random error term u does not have the first order autocorrelation. From Table3, the concomitant probability P of LM test is 0.4276, which is greater than 0.05. The hypothesis is thus accepted, which is the autocorrelation of the model does not exist at the 5% level of significance. Therefore, the revised model is tenable and not need to be revised again.

**Table 3:** The results of heteroscedasticity test and autocorrelation test for the revised model.

Method	Statistics	P
White	7.8509	0.0972
LM	0.6294	0.4276

**Figure 10:**
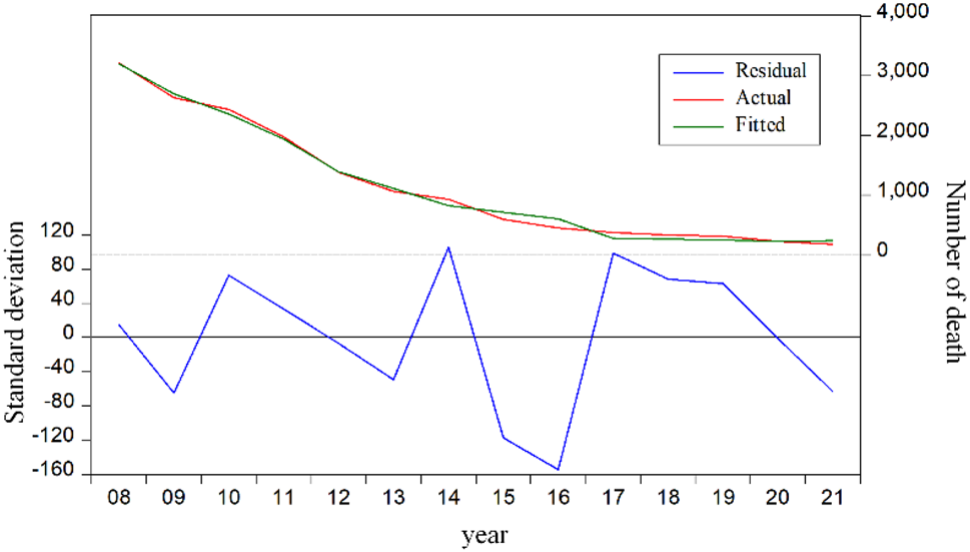
The results of residual analysis for the revised model.

Figure 10 shows the results of residual analysis for the revised model. From Fig. 9, the residual fluctuates around zero, indicating that the fitting effect of model is well.

Table 4 shows the error between the predicted number of deaths and the real value. From Table 4, the results estimated by the revised model showed a smaller error, indicating that the fitting effect of model is well.

**Table 4:** The predicted number of deaths and the real value.

Years	True death	Predicted	Error
2008	3215.000	3200.333	6.289
2009	2631.000	2696.064	− 56.747
2010	2433.000	2359.885	92.562
2011	1973.000	1939.662	66.699
2012	1384.000	1390.755	3.248
2013	1067.000	1116.301	− 50.978
2014	931.000	825.112	108.476
2015	598.000	715.331	− 119.414
2016	451.000	605.549	− 161.304
2017	375.000	276.205	78.025
2018	333.000	264.503	53.698
2019	316.000	252.802	54.372
2020	228.000	229.398	1.718
2021	178.000	241.100	− 65.955

#### Analysis of model

The regression coefficient of the revised model is 0.991; that is, 99.1% of the total deviations in the death toll can be explained by the model. The model passes the significance test, heteroskedasticity test, and autocorrelation test, indicating that the predicted results are reasonable. The regression results for the different stages are given by following equations.

1. 2008 to 2011: $$Y_{t1}^{ * } = 5608.636 - 80.679X_{t}$$
2. 2012 to 2017: $$Y_{t2}^{ * } = - 224.615 + 30.615X_{t}$$
3. 2018 to 2021: $$Y_{t3}^{ * } = 1011.200 - 22.650X_{t}$$

From 2008 to 2011, the coefficient is − 80.679, which represents the impact of safety policies on the number of deaths. Similarly, those of 2012–2017 and 2018–2021 are 30.615 and − 22.650.

### Results of impulse response analysis

Based on the index of safety policy in "Comparison between coal mine and construction industry accidents" section, the method of impulse response analysis is applied to the lag of policy implementation and its effects, as well as the effects of different types of policies. The process includes augmented dickey-fuller (ADF) test, cointegration test, VAR test, and impulse response analysis.

Table 5 shows the results of ADF test. Where, if the ADF test is greater than 5%, it is unstable, otherwise it is stable.

**Table 5:** The results of augmented dickey-fuller (ADF) test.

The order		ADF test	5%	Stability
Number of death Y	Initial order	− 6.4984	− 1.9777	Unstable
	First order difference order	–	–	–
Breadth B	Initial order	− 0.2941	− 1.9710	Unstable
	First order difference order	− 3.0951	− 1.9777	Stable
Intensity I	Initial order	− 0.7162	− 1.9710	Unstable
	First order difference order	− 4.1330	− 1.9777	Stable
Universality U	Initial order	− 0.0901	− 1.9710	Unstable
	First order difference order	− 7.9608	− 1.9823	Stable

From Table 5, the interpreted variable Y is a zero-order single integer and the explanatory variables B, I, and U are first-order single integers, indicating that a cointegration test can be performed.

Because of the small sample in this study, the EG two-step method is applied for inspection. First, OLS regression is performed and the residual is calculated. Secondly, an ADF test is performed on the residual. Tables 6 and 7 show the results of OLS regression and the ADF test for the residual.

**Table 6:** The results of OLS regression.

Variable	Coefficient	Std. error	t-statistic	Prob.
B	− 194.7703	165.5161	− 1.1767	0.2665
I	113.0084	126.3453	0.8944	0.3921
U	− 43.2799	177.2729	− 0.2441	0.8121
C	697.9764	2147.9780	0.3249	0.7519
R-squared	0.2450	Mean dependent var		1150.9290
Adjusted R-squared	0.0185	S.D. dependent var		1016.8750
S.E. of regression	1007.4410	Akaike info criterion		16.9032
Sum squared resid	10,149,372	Schwarz criterion		17.0858
Log likelihood	− 114.3222	Hannan-Quinn criter		16.8863
F-statistic	1.0815	Durbin-Watson stat		0.4789
Prob(F-statistic)	0.4007

**Table 7:** The results of the ADF test for the residual.

The order		ADF test	5%	Stability
Error	Initial order	− 2.4942	− 1.9710	Stable

From the results, there is a cointegration relationship between variables. Cointegration equation is given by Eq. (6).

6 $$Y = 697.9764 - 194.7703GD + 113.0084QD - 43.2799FY$$

It can be seen that linear combination of B, I and U is stable, and there is a linear relationship between them and the number of deaths, although the three policy indicators are not stable.

A VAR model was established using Eviews10 and Granger causality was conducted. Table 8 shows the results of Granger test. The results showed that within the range of 10% significance level, B and I were the Granger causes of the death toll, while U had no relationship with the death toll. That is changes in the B and I of policy indexes in the previous stage can lead to changes in the number of deaths, indicating that the implementation of coal mine safety policy can have effects on the number of deaths in coal mine accidents.

**Table 8:** The results of Granger test.

The hypothesis	Size of sample	F	P
B does not Granger Cause Y	12	5.5549	0.0359
Y does not Granger Cause B		2.9597	0.1171
I does not Granger Cause Y	12	14.0044	0.0036
Y does not Granger Cause I		0.0362	0.9646
U does not Granger Cause Y	12	1.9206	0.2163
Y does not Granger Cause U		3.2078	0.1026
I does not Granger Cause B	12	0.1474	0.8656
B does not Granger Cause I		5.8431	0.0322

**Figure 11:**
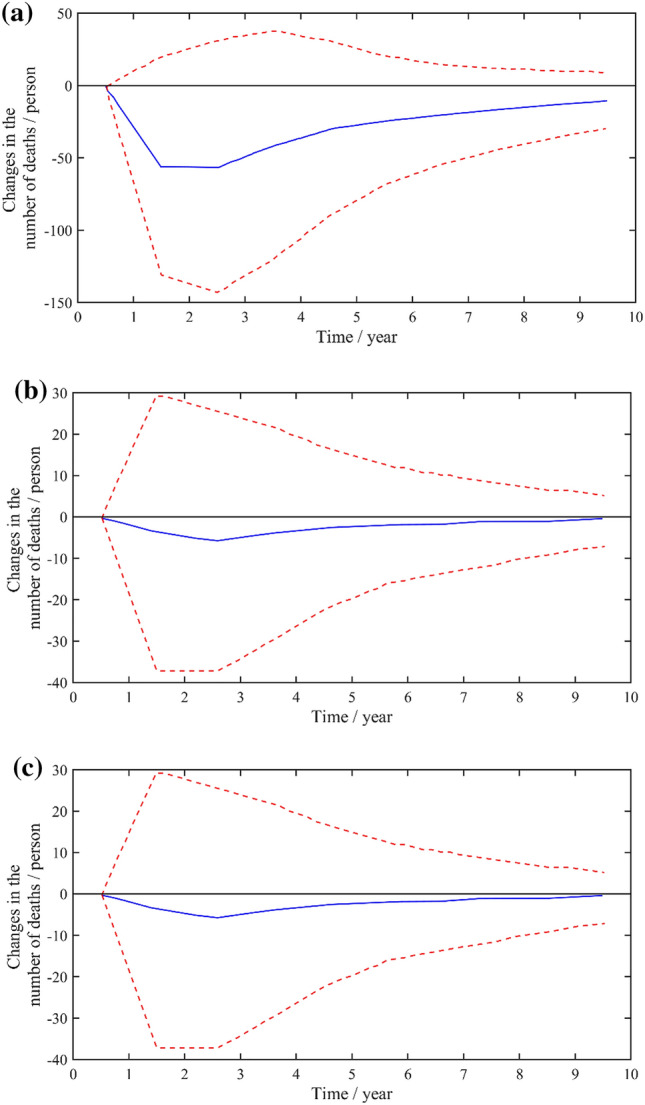
Pulse response of policy indicators to changes in the number of deaths.

Impulse response analysis was conducted. In stage t, a unit pulse is given to the B, I and U respectively, and there is no external pulse interference, using to analyze the change of the pulse response function of the number of deaths in a stage. Figure 11 shows the impulse response function. The lag period of impact is 10 years.

From Fig. 11a, the number of deaths had a negative impact on the B, reaching the maximum in the second and third stage, and then gradually decreased. Similarly, the death toll to the B shows a negative impact, reaching the maximum in the third stage. The death toll has slight impact on the U, which can be ignored. In all, the B of policy indicators has the greatest impact on the death toll, followed by the B, while the U has slight impact. Therefore, it can be seen that the impact of national policies is obviously greater than that of local policies, and the effect is more obvious with the increase of the legal effect of policies. The national regulatory policy with wider coverage and stronger system can effectively prevent the occurrence of safety accidents.

In the examination of change points, Fig. 9 illustrates notable occurrences in the years 2011, 2013, and 2017. Employing a direct segmented regression analysis led to a reduction in sample size and subsequent decrease in accuracy. Consequently, pivotal indicator dummy variables were introduced at these specific change points, namely, in 2011, 2013, and 2017.

In the exploration of lagging and leading indicators, Fig. 11 portrays the pulse response function representing the influence of policy factors on coal mine fatalities during distinct time intervals (lagging and leading). The periods spanning from the first to the third are classified as the leading phase, witnessing a continuous escalation in the adverse impact of policy factors on coal mine fatalities, culminating in its zenith during the third period. Conversely, the 3–10 period signifies a lag phase, characterized by a gradual attenuation of the negative impact. This attenuation effect eventually stabilizes, indicating a limited and consistent influence on the number of coal mine fatalities.

This analysis elucidates the more potent negative impact of policy factors on coal mine accidents within the initial three-year timeframe. Consequently, it is imperative to enact policy revisions and enhancements after this period to bolster their sustained negative impact.

## Conclusions

Statistical analysis was conducted to find out the characteristics of coal mine accidents in China based on the collected data on coal mine accidents. The results show that the number of coal mine accidents and deaths in China has shown a significant downward trend in recent years. Gas accidents account for more than half of major and above accidents. Roof and flood accidents have also been types with a high frequency, which are only lower than gas accidents. The safety policy reduced the coal mine accidents effectively in both China and America.

An ordinary least squares (OLS) regression model is established to conduct a quantitative analysis of the correlation characteristics between coal mine accidents and safety policy. The established model was then tested and revised by model fitting and significance test, heteroscedasticity test, and autocorrelation test. Finally, the quantitative relationship between the number of safety production policies and coal mine accidents is analyzed according to the revised regression model. The results show that the relationship between the policy and the number of accident deaths is progressive. Safety policies have impacts on the prevention of accidents, but the safety production situation is on the rise on the whole.

Based on the classification and analysis of China’ s safety policies, three policy indicators were identified, specifically, intensity, breadth, and universality. Unit root, cointegration, and Granger tests were conducted for the above indicators, all of which passed the various tests. By using Eviews10 to establish a VAR model and analyze the impulse response of the number of deaths to each safety policy indicator. The results show that the impact of national policies is greater than that of local policies, and the effect is more obvious with the increase in the legal effect of policies. A national regulatory policy with wider coverage and a stronger system can effectively prevent the occurrence of safety accidents.

## Data Availability

The data that support the findings of this study are available on request from the corresponding author. The data are not publicly available due to privacy or ethical restrictions.
